# Rethinking 15-min walkable neighborhoods for healthy aging: evidence from cardiovascular health in Shanghai’s older adults

**DOI:** 10.3389/fpubh.2025.1687414

**Published:** 2025-11-21

**Authors:** Cui Wu, XingZhao Liu, Yanting Xu, Yang Meng, Jing Wu

**Affiliations:** 1 Shanghai Baoshan Center of Disease Prevention and Control, Baoshan, China; 2 College of Arts and Landscape Architecture, Fujian Agriculture and Forestry University, Fuzhou, China; 3 College of Architecture and Urban Planning, Tongji University, Shanghai, China; 4 College of Architecture and Urban Planning, Fuzhou University, Fuzhou, China

**Keywords:** cardiovascular health, 15-min walkable neighborhood, cross-sectional study, older adults, health disparity

## Abstract

Interventions targeting chronic diseases through urban built environments have gained increasing attention due to their potential population-level health benefits. However, empirical evidence on the relationship between built environment features and cardiovascular health among older adults remains limited, particularly in ultradense Asian cities experiencing rapid population aging. This study analyzed fine-scale built environment data to delineate 15-min walkable neighborhoods around 2,579 stroke emergency visits among adults aged 60 to 80 in Shanghai. Participants were classified as survivors or non-survivors based on outcomes during their initial hospitalization, serving as a proxy for cardiovascular health status. Using binary logistic regression, we examined associations between neighborhood environmental characteristics and cardiovascular outcomes. Results indicated that higher residential building density and proximity to major roads were significantly linked to poorer cardiovascular health, whereas greater neighborhood greenness, measured by the Normalized Difference Vegetation Index (NDVI), was associated with better outcomes, especially within socioeconomically disadvantaged neighborhoods. Streetscape features such as visible sky and greenery were positively correlated with better cardiovascular health in more affluent areas. Additionally, a U-shaped relationship emerged between the proportion of residential land use and cardiovascular outcomes in disadvantaged communities. These findings provide nuanced, context-specific insights into how fine-scale built environment factors relate to cardiovascular health among aging urban populations, offering valuable implications for land use planning and healthy urban design.

## 1 Introduction

According to the World Health Organization, the global population aged 60 and above is projected to reach 1.4 billion by 2030, underscoring the urgent need to prioritize healthy aging as a critical public health challenge (1, 2). In China, this demographic transition is occurring at an accelerated pace, with older adults disproportionately affected by chronic diseases due to age-related physiological decline and relatively lower socioeconomic status (3). Cardiovascular diseases (CVDs), particularly stroke, represent a major health threat for older populations and have become the leading cause of death worldwide (4, 5), placing substantial burdens on healthcare systems and broader societal resources (6).

While pharmacological interventions remain the primary approach for mitigating cardiovascular risk, increasing attention has turned toward the built environment (BE) as a modifiable, non-clinical determinant of health capable of generating sustained population-level benefits through structural and behavioral pathways (7, 8). The BE encompasses human-made physical surroundings that support daily living, including land-use patterns, street connectivity, public infrastructure, and access to green spaces. A widely recognized framework for evaluating BE is the “5D” model, comprising five interrelated dimensions: density, diversity, design, destination accessibility, and distance to transit (8). These dimensions collectively shape individuals’ physical activity, travel behavior, and lifestyle choices, thereby influencing cardiovascular health outcomes (9).

Given the growing recognition of the BE as a key health determinant, it is essential to examine how specific population subgroups interact with their environments. Older adults represent a particularly vulnerable demographic whose health outcomes are highly sensitive to the quality and accessibility of their immediate surroundings. With advancing age, individuals often experience reduced mobility, declining physical capacity, and heightened sensitivity to environmental constraints. Consequently, their spatial behavior becomes more localized, and they increasingly rely on the availability and quality of nearby services, infrastructure, and amenities (10). In this context, the neighborhood environment emerges as a critical setting for promoting physical activity, social participation, and the prevention of chronic diseases such as CVD.

In response to these challenges, urban researchers and planners have increasingly developed actionable planning models that translate theoretical knowledge of the BE into practical, health-supportive interventions, particularly tailored to aging populations. Neighborhood-based spatial planning models emphasizing proximity, accessibility, and walkability have attracted substantial attention. These models operationalize the abstract dimensions of the 5D framework into tangible urban forms that shape residents’ daily experiences, especially for individuals with reduced mobility. This discourse has culminated in the emergence of the “15-min walkable neighborhood” concept—a promising planning paradigm integrating health, equity, and accessibility. Endorsed by urban policies worldwide, the 15-min walkable neighborhood aims to ensure essential services such as healthcare, public transport, recreational facilities, and daily amenities are accessible within a 15-min walk (11–14). By supporting active travel and facilitating routine engagement with one’s environment, this model promotes physical activity, social interaction, and beneficial environmental exposures, which are especially vital for older adults’ health and well-being (15). As such, the 15-min neighborhood holds considerable potential to mitigate spatial isolation, reduce health disparities, and improve cardiovascular outcomes among urban aging populations (16).

Recently, the 15-min neighborhood framework has gained traction in Chinese cities as part of broader strategies to advance public health and sustainable urban development (15). Against this backdrop, the present study investigates associations between fine-grained BE characteristics and cardiovascular health among older adults residing within 15-min walkable neighborhoods in urban China (17, 18). Leveraging high-resolution spatial data, we examine the neighborhood environments of 2,579 Shanghai residents aged 60 to 80. Situated within a densely populated Asian megacity, this study provides novel empirical evidence on spatial mechanisms linking neighborhood-level BE features to health outcomes in later life. Moreover, by incorporating an equity perspective, it explores how these associations vary across neighborhoods with differing socioeconomic profiles, thereby informing both theoretical development and practical strategies for inclusive, health-oriented urban planning.

## 2 Literature review

Existing studies have investigated the complex relationships between various domains of neighborhood built environment characteristics and cardiovascular health, including land use, open spaces, transportation infrastructure, service facilities, and streetscape features (see Table 1). The built environment influences cardiovascular health primarily through two key pathways (19). The first involves prolonged exposure to environmental stressors such as air pollution and traffic noise, which can disrupt physiological processes by elevating blood pressure and altering lipid and glucose metabolism, thereby increasing cardiovascular risk (20–22). For example, urban roads are major sources of air and noise pollution closely linked to elevated cardiovascular morbidity and mortality (23). The second pathway operates through behavioral and social mechanisms. Access to green spaces supports cardiovascular health by promoting physical activity and facilitating social interactions (24, 25). Moreover, higher land-use mix and greater road connectivity within neighborhoods can reduce travel distances, encourage walking or cycling, and foster more active lifestyles, ultimately contributing to improved cardiovascular health outcomes (26, 27). Collectively, these findings highlight the crucial role of neighborhood built environments in shaping health-related behaviors and mitigating harmful exposures, underscoring their potential as effective targets for public health interventions in urban settings. These mechanisms are central to emerging urban planning concepts such as the 15-min walkable neighborhood, which aim to align everyday accessibility with health-supportive environments.

**Table 1:** Existing findings about the association between the built environment and cardiovascular health.

Dimension	Built environment variables	Correlation with cardiovascular health
Land use	Population density	(29) (−); (30) (~); (28)(+)
	Building density	(28) (+); (74) (~)
	Floor area ratio	(75) (+); (28) (N)
	Residential land/density	(30)(~); (28) (+)
	Land mixture	(30) (+); (28) (N); (30) (~)
	Business land	Hypothesis in the current study (+)
	Industrial land	(29) (+); (28) (N); (32) (+)
Open space	NDVI/ green space/park	(76) (−); (42) (−); (75)(N); (74) (~); (77) (−)
Transportation	Bus or metro station	(75) (+); (30) (N); (75)(+)
	Distance to the nearest major road	(78) (+); (79) (+)
	Road intersection density	(80) (+); (29)(~); (28) (N)
	Road density/street connectivity	(30)(+); (28) (N); (32) (−); (80) (N);
	Sports facility density	(13) (−); (75) (−); (81) (−);
	Healthy food facilities	(13) (−); (29) (~); (75) (−); (82) (−)
	Unhealthy food facilities	(82) (+); (28) (N); (83) (+);
	Medical facility density	(29) (−); (75)(N); (45) (−); (28) (−)
Streetscape	Greenery	(33) (−); (46) (−)
	Sky	Hypothesis in the current study (+)
	Sidewalk	Hypothesis in the current study (+)

“(+)” indicates a positive correlation; “(−)” indicates a negative correlation; “(N)” indicates no significant correlation; and “(~)” indicates a non-linear (e.g., curvilinear) association. NDVI, Normalized Difference Vegetation Index. Healthy food facilities include supermarkets, food stores, fruit vendors, and vegetable vendors. Unhealthy food facilities include processed food vendors, take-away outlets, fast-food restaurants, and snack bars.

Despite increasing research interest, critical gaps remain in our understanding of the built environment–cardiovascular health nexus. First, empirical findings have been inconsistent (see Table 1). For instance, some studies report a negative association between population density and cardiovascular mortality (28, 29), while others observe an inverted U-shaped relationship or no significant effects (6, 30).

Similar inconsistencies arise concerning land-use mixture and road density (30, 31). These discrepancies may stem from imprecise neighborhood definitions and reliance on coarse spatial measurements, limiting deeper insights into the complex interplay between built environment attributes and health outcomes (28, 32). Second, several potentially important built environment factors remain underexplored, particularly street-level characteristics within walkable areas for older adults (see Table 1). Urban streets, as essential settings for daily activities such as walking and cycling, may directly or indirectly influence residents’ behaviors and health outcomes (33, 34). Positive street elements, such as blue skies, tree shade, and wide, well-maintained sidewalks, can enhance perceived comfort and safety, thereby encouraging outdoor physical activity and supporting cardiovascular health (34, 35).

Although some research acknowledges the role of green visibility, the health implications of other street-level features remain unclear. Third, structural barriers prevalent in many metropolitan areas—such as spatial mismatch, social inequality, and insufficient age-friendly planning—continue to hinder equitable access to health-supportive environments (36–39). These challenges disproportionately affect older adults, who are more vulnerable due to age-related functional decline and a higher dependency on proximate services. Older individuals living in socioeconomically disadvantaged neighborhoods often face limited access to essential amenities such as healthcare, recreational spaces, and commercial facilities, which restricts opportunities for health-promoting behaviors and exacerbates disparities in cardiovascular outcomes (40–43).

To address these challenges, the present study focuses on older adults residing in large urban areas and examines the relationship between detailed built environment characteristics and cardiovascular health within the framework of 15-min walkable neighborhoods. By concentrating on this high-risk population, the study aims to provide empirical evidence to inform inclusive, health-oriented urban planning strategies that promote active aging and reduce spatial health inequalities.

## 3 Materials and methods

### 3.1 Research framework

**Figure 1:**
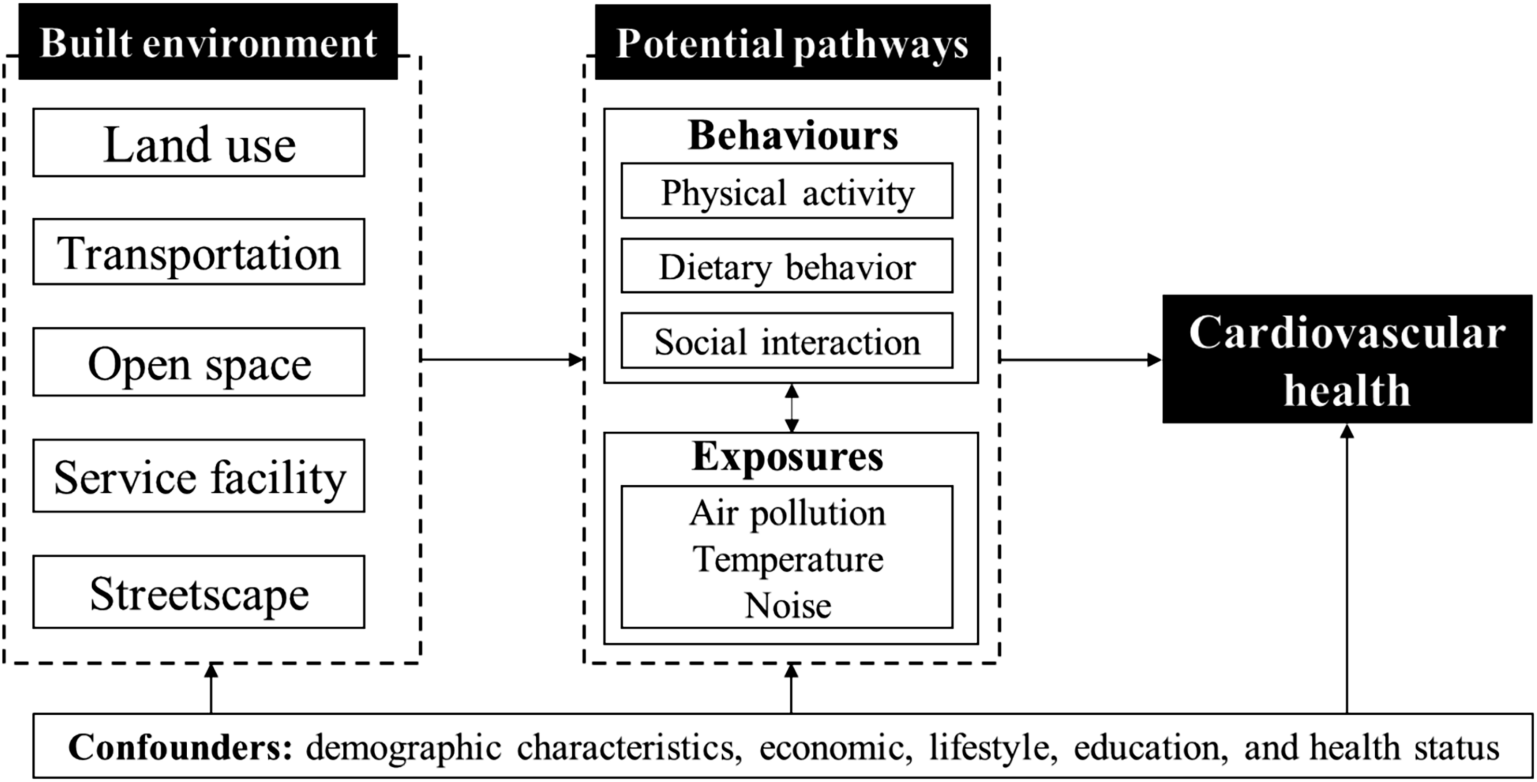
Research framework of the built environment on cardiovascular health.

As previously noted, the pathways through which the built environment influences cardiovascular health can be broadly categorized into exposure-related and behavior-related mechanisms. Exposure-related pathways involve contact with harmful environmental factors such as air pollution and noise, while behavior-related pathways encompass lifestyle factors including diet, physical activity, and social interaction. To conceptualize these mechanisms, we developed a comprehensive analytical framework focusing on the built environment at the scale of a 15-min walkable neighborhood. This framework incorporates five key dimensions: land use, transportation, open space, service facilities, and streetscape features (see Figure 1). We hypothesize that variables within each dimension impact cardiovascular health through these dual pathways.

### 3.2 Study area and data resource

Shanghai is recognized as a leading global center for economic, financial, trade, shipping, and technological innovation in China. In 2021, the city’s gross domestic product (GDP) reached 4.32 trillion yuan, ranking first among all Chinese cities. This study selects Baoshan District as the empirical research area. Although Baoshan is a suburban district of Shanghai, its population size (about 2.1 million) is comparable to that of many medium-sized cities. In 2019, its population density (3,437 persons/km^2^) was roughly twice that of Portland (1,684 persons/km^2^) (44). Meanwhile, the district is experiencing pronounced population aging, with residents aged 60 and above accounting for 41.1% of the total population by 2023. The intersection of high population density and an increasingly older adults population may contribute to elevated cardiovascular risks, making Baoshan a representative case for exploring the relationship between the built environment and cardiovascular health.

Given the high prevalence of stroke among the older adults and its well-documented sensitivity to environmental risk factors, this study categorized participants as survivors or non-survivors based on their initial stroke admission outcomes, which served as a proxy for cardiovascular health status. This approach has been employed in some studies examining the relationship between the built environment and cardiovascular disease (45, 46). A binary health outcome variable was therefore constructed to represent initial stroke severity: “0” denoting survival and “1” denoting death at the initial onset of stroke. Daily stroke incidence data in 2018 were obtained from the Hospital Quality Monitoring System (HQMS) in Baoshan District. This comprehensive surveillance system captures patient admissions across all levels of medical institutions, including private clinics, community health centers, and public hospitals, ensuring wide coverage and high data reliability. The dataset includes critical patient-level information such as age, gender, date of death, and residential address.

**Figure 2:**
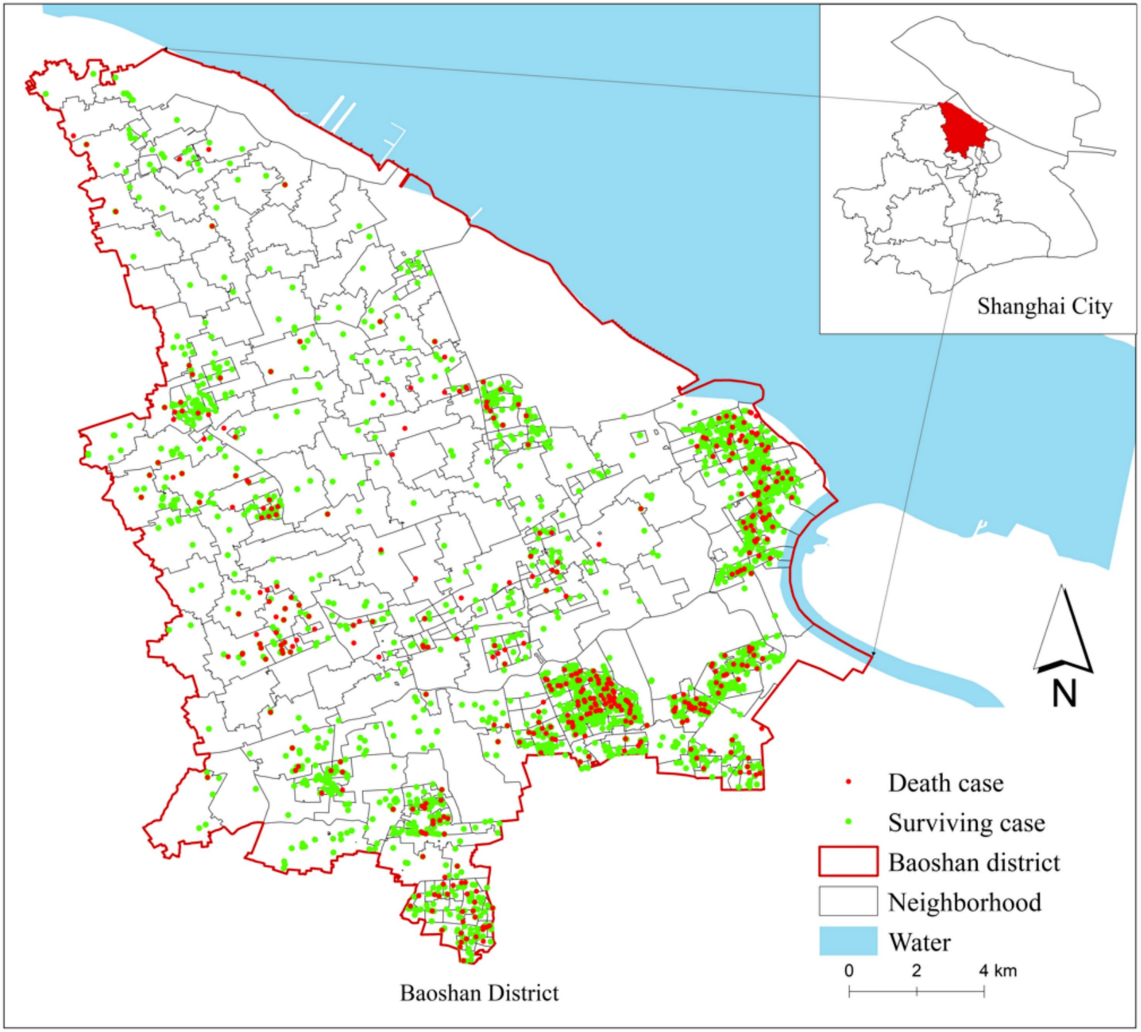
Residential location of patients (*n* = 2,579).

To align with the study’s focus on older adults at elevated cardiovascular risk, the sample was restricted to long-term residents of Baoshan District aged 60–80 years. In determining the minimum residence duration, we referred to previous longitudinal studies examining the relationship between built environment and cardiovascular outcomes. Specifically, a Dutch study based on the Obesogenic Built-environmental Characteristics (OBCT) cohort followed participants for 14 years (47), while a longitudinal study in London, UK reported an average follow-up of 8.4 years (48). Accordingly, we defined the minimum residence period in our study as the average of these durations, i.e., 11 years, ensuring that participants had sufficient long-term exposure to the local environment to plausibly influence cardiovascular health. After applying these criteria, the final dataset included 2,579 anonymized stroke cases. Residential addresses were geocoded using the Baidu Map API to support spatial analysis (see Figure 2).

### 3.3 Built environment variables

**Figure 3:**
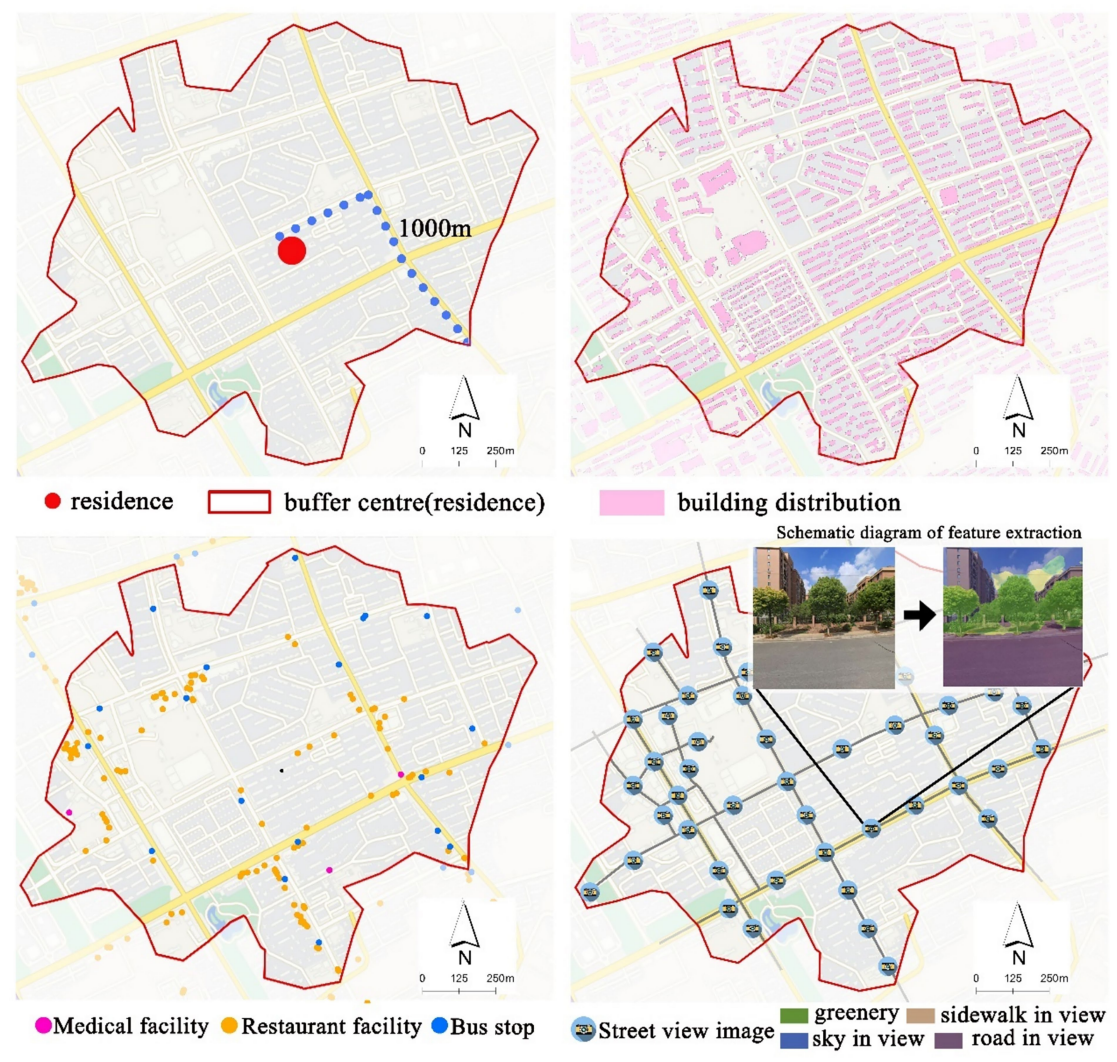
Definition of the 15-min walkable neighborhood and measurement of the built environment.

Based on residential addresses, we were able to pinpoint the locations of participants on the map using ArcGIS Geocoding tool. To define 15-min walkable neighbourhoods for older adults, we created 1,000-meter street-based network buffers around each residence using ArcGIS Network Analyst (see Figure 3). The network buffer represents the area reachable within 15 min based on actual walking paths, excluding non-walkable roads such as highways. Older adults typically walk at 48–60 meters per minute (49), making 1,000 meters a reasonable estimate for a 15-min walk. Compared to circular buffers or administrative boundaries, this method more accurately reflects the built environment people experience in daily life (12, 17).

We selected built environment variables known to influence residents’ behavior and environmental exposure (19, 50), following the guidelines set by the Shanghai Planning Guidance for the 15-min Community-Life Circle. A total of 19 variables across five categories were included, with detailed definitions and calculation methods provided in Table 2. Data sources included point of interest (POI) data for service facilities from Baidu Map,^1^ vector road network data from OpenStreetMap^2^ as of 2018, and current land use and building data (2018) from the Shanghai Land Resources & Planning Information Center. Additionally, street view greenery was incorporated to represent residents’ perceived street-level environmental quality (33).

**Table 2:** Summary statistics for all variables.

Variables		Definition and calculation	Total (*n* = 2,579)	Survive (*n* = 2,317)	Dead (*n* = 262)
			Mean (SD)	Mean (SD)	Mean (SD)
Control variable					
Age (year)		Patient information	69.872 (0.117)	69.803 (0.123)	70.485 (0.379)
Male			1,503	1,329 (88%)	174 (12%)
Female			1,076	988 (91%)	88 (8%)
Neighborhood deprivation index		A comprehensive measure of deprivation, reflecting poverty, employment, housing, education, and demographics at the neighborhood level.	0.611 (0.033)	0.594 (0.034)	0.762 (0.108)
Distance to emergency departments (m)		Distance between patient’s residence and emergency departments based on actual streets.	1305.341 (15.280)	1315.084 (16.366)	1219.185 (40.613)
Independent variables					
Land use	Population density (people/m^2^)	The population of the participant’s neighbourhood was used.	3.500 (0. 000)	3.543 (0. 000)	3.566 (0.000)
	Building density (%)	The ratio of the total base area of the buildings in the street-based network buffer to the total area of the street-based network buffer.	19.800 (0.100)	19.700 (0.100)	20.100 (0.300)
	Land Mixture	The land-use mixture of the participant’s neighbourhood was used.	0.063 (0.002)	0.065 (0.002)	0.053 (0.005)
	Residential land (%)	The commercial, residential, and industrial areas within the street-based network buffer are each calculated as a proportion of the total area of the street-based buffer.	56.818 (0.502)	56.179 (0.533)	62.468 (1.439)
	Industrial land (%)		13.552 (0.435)	13.99 (0.467)	9.681 (1.115)
	Commercial land (%)		2.885 (0.153)	2.899 (0.163)	2.753 (0.429)
Transportation	Road density (km/km^2^)	The road density was calculated as the total length of roads divided by the total area of the street-based buffer, primarily including major and minor roads. According to the official “China Major Cities Road Network Density Monitoring Report (2018),” a value of 8.0 km/km^2^ is considered the target standard for modern urban development (84).	12.300 (1.500)	12.200 (1.400)	12.800 (1.600)
	Number of metro stations (n)	The total number of metro stations within the street-based network buffer.	0.390 (0.110)	0.380 (0.110)	0.490 (0.037)
	Distance to main roads (m)	It refers to the Euclidean (straight-line) distance from the patient’s residence to the nearest motorway or major road, calculated using GIS software based on geocoded residential addresses and road network data.	280.87 (4.962)	283.047 (5.320)	261.620 (13.102)
Service facility	Restaurant density (n/m^2^)	The total number of restaurants, medical facilities, and bus stops is divided by the total area of the street-based buffer.	0.099 (0.015)	0.102 (0.016)	0.069 (0.034)
	Bus stop density (n/m^2^)		0.103 (0.007)	0.104 (0.007)	0.101 (0.021)
	Medical density (n/m^2^)		0.060 (0.002)	0.060 (0.002)	0.064 (0.008)
Open space	Average NDVI	The average of NDVI values of 30 × 30 m grids within the street-based network buffer.	0.140 (0.004)	0.136 (0.004)	0.174 (0.013)
	Distance to public park (m)	Distance between patient’s residence and nearest parks based on actual streets.	581.954 (8.544)	585.927 (9.086)	546.814 (24.793)
Streetscape	Sidewalk	We captured street view images (at 0°, 90°, 180°, and 270°) from Tencent Map at 100-meter intervals along the road network. The sidewalk, greenery, and sky proportions were then calculated by averaging all sample points within the street-based network buffer (85, 86).	0.021 (0.004)	0.020 (0.003)	0.022 (0.005)
	Greenery		0.201 (0.001)	0.200 (0.001)	0.205 (0.003)
	Sky		0.189 (0.001)	0.188 (0.001)	0.193 (0.004)

### 3.4 Covariates

Due to the unavailability of individual-level data on income and education, we used a neighborhood deprivation index to represent socioeconomic status as a potential confounder (51, 52). This index captures material and social deprivation across five domains: poverty, employment, housing, education, and demographic characteristics. Following the approaches of (53, 54), we obtained community-level indicators from the 6th Shanghai National Census (2010), including the proportion of low-income residents, the proportion of unemployed residents, the proportion of individuals with no formal education, the proportion of households with a residential building area below 29 m^2^ (the smallest category in the official classification), and the proportion of residents without local household registration (55). These indicators were integrated using principal component analysis (PCA) to construct the neighborhood deprivation index, which was subsequently categorized into tertiles for stratified analysis: tertile 1 (least deprived), tertile 2 (moderately deprived), and tertile 3 (most deprived). All models were further adjusted for gender, age, and distance to the nearest emergency department, given their potential influence on cardiovascular outcomes. A summary of all variables is provided in Table 2.

### 3.5 Statistical analysis

To address multicollinearity, we excluded variables with a variance inflation factor (VIF) greater than 3. We then employed binary logistic regression models to examine the association between built environment characteristics within 15-min walkable neighborhoods and initial stroke severity (0 = survival, 1 = death). Model 1a assessed the overall population. Models 2a and 2b conducted stratified analyses by gender, while Models 3a, 3b, and 3c examined effects across tertiles of neighborhood deprivation (least-, medium-, and most-deprived). All models controlled for age, gender, and distance to the nearest emergency department. Two-tailed *p*-values and 95% confidence intervals were calculated, and all analyses were performed using SPSS version 22.

The general form of the logistic regression model used is:


$$\text{Logit}(P(Y=1))=\beta_{0}+\beta_{1}X_{1}+\beta_{2}X_{2}+\ldots \ldots +\beta_{k}X_{k}$$

Where *Y* represents initial stroke severity (0 = survival, 1 = death), *X*_1_, *X*_2_,……, *X*_k_ denote covariates including built environment characteristics, age, gender, and distance to the nearest emergency department, and *β*_0_, *β*_1_, ……, *β*_k_ are the regression coefficients.

## 4 Results

Table 3 summarizes the associations between built environment characteristics within 15-min walkable neighborhoods and stroke mortality, both for the overall sample and stratified by gender. The results show that older adults residing in areas with moderate building density (22.6–37.6%) and a higher proportion of industrial land use (0.3–9.9%) had an increased likelihood of stroke-related mortality (OR = 1.397, 95% CI: 0.971–2.008; and OR = 1.573, 95% CI: 0.997–2.479, respectively). A greater number of metro stations was also associated with elevated stroke mortality risk (OR = 1.480, 95% CI: 1.166–1.879). In contrast, higher levels of greenness, as measured by the average Normalized Difference Vegetation Index (NDVI) (0.002–0.196), were significantly associated with lower odds of stroke mortality (OR = 0.650, 95% CI: 0.488–0.866). Regarding streetscape features, areas with moderate sidewalk coverage (0.023–0.049) showed a positive association with stroke mortality (OR = 1.483, 95% CI: 1.043–2.107). Conversely, greater visual exposure to greenery and sky demonstrated potential protective effects (OR = 0.705, 95% CI: 0.472–1.053; and OR = 0.660, 95% CI: 0.436–0.997, respectively). These associations were generally more pronounced among male participants, suggesting possible gender-specific sensitivities to built environment influences on cardiovascular health.

**Table 3:** The association between the built environment within the 15-min neighborhood and stroke mortality for all populations and stratified by gender.

Variables		Model 1	Model 2a	Model 2b
		OR (95% CI)	OR (95% CI)	OR (95% CI)
Covariates	Age	1.020 (0.998, 1.043)	1.017 (0.989, 1.046)	1.031 (0.991, 1.072)
	Gender	1.514** (1.148, 1.997)	—	—
	Neighborhood deprivation index	1.066 (0.959, 1.184)	1.057 (0.929, 1.204)	1.060 (0.874, 1.286)
	Distance to emergency departments	0.750 (0.439, 1.280)	0.631 (0.326, 1.222)	1.197 (0.458, 3.131)
Independent variables				
Land use	Population density	1.115 (0.518, 2.399)	1.150 (0.434, 3.046)	1.528 (0.422, 5.532)
	Building density (ref: Q1)	1	1	1
	Q2(18.7–22.5%)	1.407 (0.867, 2.283)	1.433 (0.777, 2.645)	1.428 (0.627, 3.251)
	Q3(22.6–37.6%)	1.397* (0.971, 2.008)	1.586** (1.006, 2.498)	1.134 (0.601, 2.140)
	Land mixture	0.345 (0.069, 1.724)	0.484 (0.069, 3.409)	0.119 (0.006, 2.225)
	Percentage of commercial land (ref: Q1)	1	1	1
	Q2 (0.1–0.6%)	0.842 (0.600, 1.181)	0.824 (0.536, 1.267)	0.812 (0.453, 1.456)
	Q3 (0.7–59.2%)	1.005 (0.549, 1.839)	1.329 (0.666, 2.65)	0.337 (0.073, 1.552)
	Percentage of residential land (ref: Q1)	1	1	1
	Q2 (50.9–71.1%)	0.784 (0.477, 1.287)	0.752 (0.403, 1.402)	0.868 (0.360, 2.092)
	Q3 (71.2–96.4%)	0.966 (0.662, 1.410)	1.021 (0.638, 1.634)	0.842 (0.423, 1.676)
	Percentage of industrial land (ref: Q1)	1	1	1
	Q2 (0.3–9.8%)	1.573*(0.997, 2.479)	1.406 (0.79, 2.502)	1.975 (0.893, 4.366)
	Q3 (9.9–10%)	1.069 (0.701, 1.63)	1.062 (0.625, 1.803)	1.046 (0.493, 2.220)
Transportation	Road density (ref: Q1)	1	1	1
	Q2 (11–12.7%)	1.172 (0.743, 1.848)	1.186 (0.671, 2.096)	1.152 (0.522, 2.542)
	Q3 (12.8–26%)	1.012 (0.693, 1.478)	1.245 (0.78, 1.987)	0.710 (0.361, 1.397)
	Number of metro stations	1.480*** (1.166, 1.879)	1.691*** (1.252, 2.283)	1.136 (0.745, 1.733)
	Distance to main roads	1.011 (0.741, 1.381)	1.418 (0.932, 2.157)	0.593** (0.367, 0.958)
Service facility	Restaurant density	0.937 (0.753, 1.167)	0.957 (0.731, 1.253)	0.861 (0.575, 1.288)
	Bus stop density	0.950 (0.638, 1.415)	0.858 (0.504, 1.459)	1.186 (0.635, 2.215)
	Medical density	1.403 (0.437, 4.506)	1.907 (0.448, 8.111)	0.938 (0.113, 7.785)
Open space	Average NDVI (ref: Q1)	1	1	1
	Q2 (0.002–0.196)	0.650** (0.488, 0.866)	0.585*** (0.409, 0.836)	0.840 (0.510, 1.385)
	Q3 (0.197–0.757)	0.541 (0.124, 2.354)	0.386 (0.049, 3.063)	0.875 (0.103, 7.407)
	Distance to public park	0.885 (0.587, 1.334)	0.907 (0.535, 1.539)	0.857 (0.435, 1.690)
Streetscape	Sidewalk in streetscape (ref: Q1)	1	1	1
	Q2 (0.016–0.022)	1.243 (0.843, 1.833)	1.442 (0.877, 2.369)	0.913 (0.475, 1.755)
	Q3 (0.023–0.049)	1.483** (1.043, 2.107)	1.809*** (1.165, 2.809)	1.003 (0.542, 1.856)
	Greenery in streetscape (ref: Q1)	1	1	1
	Q2 (0.178–0.233)	0.705* (0.472, 1.053)	0.810 (0.485, 1.353)	0.550 (0.282, 1.075)
	Q3 (0.234–0.420)	0.851 (0.603,1.203)	1.027 (0.66, 1.598)	0.573 (0.317, 1.037)
	Sky in streetscape (ref: Q1)	1	1	1
	Q2 (0.156–0.200)	0.660** (0.436, 0.997)	0.633(0.377, 1.062)	0.765 (0.369, 1.587)
	Q3 (0.201–0.423)	0.706* (0.486, 1.025)	0.696 (0.437, 1.108)	0.722 (0.375, 1.390)
Model parameter	-2 Log Likelihood	1616.100	1011.220	573.601
	Cox & Snell *R*^2^	0.028	0.039	0.032
	Nagelkerke *R*^2^	0.057	0.077	0.074

Model 1 for all population, Model 2a for male, and Model 2b for female.

*** indicates *p* < 0.001; ** indicates *p* < 0.01; * indicates *p* < 0.05.

Table 4 presents the associations between built environment characteristics and stroke mortality among older adults, stratified by neighborhood deprivation tertiles. In least-deprived neighborhoods, a moderate proportion of sidewalks in the streetscape (0.023–0.049) was significantly associated with an increased risk of stroke mortality (OR = 2.583, 95% CI: 1.039–6.425; OR = 2.694, 95% CI: 1.020–7.111). In contrast, greater visual exposure to greenery and sky was associated with a lower risk, although not all associations reached statistical significance (OR = 0.945, 95% CI: 0.313–2.857; OR = 0.270, 95% CI: 0.059–1.242). In moderately deprived neighborhoods, higher road density (approximately 11–12.7 km/km^2^) and a greater number of metro stations were linked to significantly increased stroke mortality risk (OR = 3.506, 95% CI: 1.438–8.549; OR = 1.587, 95% CI: 1.000–2.519). Conversely, greater streetscape greenery was associated with a reduced risk of stroke mortality (OR = 0.414, 95% CI: 0.186–0.922). In most-deprived neighborhoods, higher building density was positively associated with stroke mortality, while a higher average NDVI was linked to a lower risk. Additionally, residential land coverage between 50.9 and 71.1% was significantly associated with reduced stroke mortality (OR = 0.399, 95% CI: 0.173–0.920). In contrast, residential land coverage above 71.2% showed a non-significant trend toward increased risk (OR = 1.024, 95% CI: 0.597–1.755).

**Table 4:** The heterogeneous effects of the built environment within the 15-min neighbourhood and initial stroke severity stratified by neighbourhood deprivation index.

Variables		Model 3a	Model 3b	Model 3c
		OR (95% CI)	OR (95% CI)	OR (95% CI)
Covariates	Age	0.986 (0.931, 1.045)	1.049* (1.003, 1.096)	1.019 (0.988, 1.051)
	Gender	1.589 (0.802, 3.146)	1.645 (0.973, 2.781)	1.497** (1.012, 2.214)
	Distance to emergency departments	1.521 (0.237, 9.755)	0.593 (0.229, 1.533)	1.202 (0.507, 2.850)
Independent variables				
Land use	Population density	1.319(0.225,7.732)	2.755(0.603,12.589)	0.932(0.253,3.428)
	Building density (ref: Q1)	1	1	1
	Q2 (18.7–22.5%)	0.827 (0.219, 3.113)	0.833 (0.347, 1.996)	3.011** (1.398, 6.484)
	Q3 (22.6–37.6%)	2.154 (0.594, 7.815)	1.308 (0.605, 2.826)	1.526*** (0.931, 2.503)
	Land mixture	0.364 (0.283, 0.445)	0.253 (0.008, 7.539)	0.066 (0.006, 0.791)
	Percentage of commercial land (ref: Q1)	1	1	1
	Q2 (0.1–0.6%)	0.696 (0.275, 1.758)	0.888 (0.448, 1.761)	0.678 (0.403, 1.144)
	Q3 (0.7–59.2%)	1.185 (0.185, 7.604)	0.944 (0.218, 4.086)	0.862 (0.402, 1.845)
	Percentage of residential land (ref: Q1)	1	1	1
	Q2 (50.9–71.1%)	0.598 (0.159, 2.256)	1.389 (0.531, 3.633)	0.399** (0.173, 0.920)
	Q3 (71.2–96.4%)	0.719 (0.263, 1.963)	0.638 (0.270, 1.506)	1.024** (0.597, 1.755)
	Percentage of industrial land (ref: Q1)	1	1	1
	Q2 (0.3–9.8%)	1.906 (0.486, 7.471)	1.387 (0.579, 3.324)	0.915 (0.432, 1.939)
	Q3 (9.9–100%)	2.628 (0.841, 8.209)	1.169 (0.521, 2.623)	0.506 (0.262, 0.974)
Transportation	Road density (ref: Q1)	1	1	1
	Q2 (11.0–12.7)	0.740 (0.131, 4.167)	3.506** (1.438, 8.549)	0.687 (0.346, 1.362)
	Q3 (12.8–26.0)	1.300 (0.217, 7.809)	1.345 (0.601, 3.011)	0.751 (0.461, 1.224)
	Number of metro stations	1.517 (0.760, 3.027)	1.587* (1.000, 2.519)	1.529 (1.091, 2.142)
	Distance to main roads	1.450 (0.638, 3.293)	0.810 (0.474, 1.385)	1.052** (0.646, 1.713)
Service facility	Restaurant density	1.120 (0.790, 1.589)	1.050 (0.763, 1.445)	0.185 (0.011, 3.253)
	Bus stop density	2.480 (1.189, 5.173)	0.884 (0.380, 2.052)	0.630 (0.322, 1.232)
	Medical density	0.251 (0.013, 4.815)	2.747 (0.271, 27.829)	2.171 (0.434, 10.871)
Open space	Average NDVI (ref: Q1)	1	1	1
	Q2(0.002–0.196)	0.825 (0.407, 1.676)	0.769 (0.455, 1.301)	0.575** (0.381, 0.869)
	Q3(0.197–0.757)	0.000 (0.000, 0.000)	0.963 (0.098, 9.490)	0.468*** (0.058, 3.801)
	Distance to public park	0.924 (0.274, 3.119)	1.444 (0.613, 3.399)	0.683 (0.391, 1.194)
Streetscape	Sidewalk in streetscape (ref: Q1)	1	1	1
	Q2 (0.016–0.022)	2.583* (1.039, 6.425)	1.295 (0.588, 2.854)	0.910 (0.487, 1.700)
	Q3 (0.023–0.049)	2.694* (1.020, 7.111)	1.642 (0.845, 3.194)	1.247 (0.725, 2.146)
	Greenery in streetscape (ref: Q1)	1	1	1
	Q2 (0.178–0.233)	0.945* (0.313, 2.857)	0.414* (0.186, 0.922)	0.788 (0.414, 1.499)
	Q3 (0.234–0.420)	0.932 (0.351,2.477)	0.550 (0.288, 1.051)	1.072 (0.634, 1.813)
	Sky in streetscape (ref: Q1)	1	1	1
	Q2 (0.156–0.200)	0.270* (0.059, 1.242)	0.979 (0.425, 2.257)	1.065 (0.548, 2.070)
	Q3 (0.201–0.423)	0.245 (0.080, 0.748)	1.309 (0.658, 2.607)	0.813 (0.459, 1.442)
Model parameter	−2 Log Likelihood	274.971	459.253	474.684
	Cox & Snell *R*^2^	0.070	0.055	0.050
	Nagelkerke R^2^	0.157	0.112	0.110

Model 3a for the least-deprived neighbourhoods, Model 3b for the medium-deprived neighbourhoods, and Model 3c for the most-deprived neighbourhoods.

*** indicates *p* < 0.001; ** indicates *p* < 0.01; * indicates *p* < 0.05.

## 5 Discussion

This study systematically examined the impact of micro-scale built environment characteristics on cardiovascular health among older adults, thereby contributing to the limited evidence on modifiable environmental determinants of cardiovascular outcomes in aging populations. Importantly, by incorporating neighborhood socioeconomic heterogeneity, our findings offer nuanced insights into the complex mechanisms through which neighborhood environments shape cardiovascular health in urban contexts.

A key finding of this study is the significant positive association between higher residential building density and poor cardiovascular health outcomes, including increased stroke mortality, particularly pronounced in socioeconomically disadvantaged neighborhoods. This likely reflects the compounded negative effects of overcrowding, limited access to natural and recreational spaces, and generally poorer environmental quality—factors that collectively contribute to worsening cardiovascular health (28, 31). Overcrowded living conditions can elevate chronic stress levels and reduce opportunities for physical activity, while insufficient green and open spaces limit engagement in health-promoting behaviors such as walking and social interaction. Moreover, poorer environmental quality often results in higher exposure to air pollution and noise, which are well-known to impair cardiovascular function through mechanisms like elevated blood pressure, systemic inflammation, and endothelial dysfunction (56). These combined stressors create an unfavorable environment that exacerbates poor cardiovascular health among vulnerable older adults. Notably, our findings diverge from many Western studies where moderate residential density often correlates with health benefits by promoting walkability and social interaction (57). Instead, the results align more closely with emerging evidence from other Asian megacities (58, 59), suggesting that extremely high residential densities may impose health burdens on vulnerable populations such as older adults. This discrepancy may stem from differences in urban form, infrastructure quality, and social environment, underscoring the importance of context-specific investigations.

Additionally, our study identified a non-linear, U-shaped relationship between the proportion of residential land use and stroke mortality in socioeconomically disadvantaged neighborhoods. Specifically, stroke risk was lower when residential land use accounted for less than approximately 71.2% of total land area; beyond this threshold, the risk began to increase. This pattern suggests that a balanced land-use mix may facilitate greater accessibility to diverse destinations that encourage walking and active lifestyles (30). Conversely, excessive residential land use likely reflects land-use homogenization, which reduces functional diversity and limits residents’ exposure to environments conducive to physical activity and mental well-being (60). Our findings align with prior research from Kuala Lumpur reporting similar non-linear associations between land-use composition and cardiovascular outcomes, highlighting land-use diversity as a key feature in health-oriented neighborhood planning (61). This non-linear association underscores the importance of considering not just the quantity but also the composition and configuration of land uses when designing urban environments for aging populations. Policies that promote mixed-use development—integrating residential, commercial, recreational, and green spaces—may enhance active living and reduce stroke risk, especially in deprived areas where environmental and social vulnerabilities are more pronounced.

Our study highlights the significant protective effects of neighborhood greenness on cardiovascular health, particularly among socioeconomically disadvantaged populations. These findings underscore the critical role of green spaces in promoting cardiovascular health through multiple pathways, including the encouragement of physical activity, mitigation of psychological stress, and enhancement of air quality (62, 63). Furthermore, we found that greater street visibility of sky and greenery was associated with improved cardiovascular health, especially in socioeconomically advantaged neighborhoods. Importantly, our results demonstrate that neighborhood greenness, as quantified by average NDVI, supports the Equigenesis hypothesis, suggesting that environmental improvements yield disproportionately greater cardiovascular health benefits for socioeconomically disadvantaged groups (64, 65). Conversely, streetscape greenery measured as visible green exposure at the street level did not follow this pattern. Instead, its protective effects on cardiovascular health were more pronounced in affluent neighborhoods, diverging from findings reported by Wang et al. in Guangzhou, where street-level greenery conferred stronger benefits for disadvantaged groups (33). These observations suggest that different types of green exposure, namely, macro-scale neighborhood vegetation versus micro-scale streetscape features, may influence cardiovascular health through distinct mechanisms and are experienced differently across socioeconomic groups (66, 67).

In contrast to much of the existing literature, the present study identified a positive association between the density of metro stations within a 15-min walking radius and poorer cardiovascular health outcomes. This counterintuitive finding may be attributable to the distinctive characteristics of transit-oriented development in Chinese megacities (68), where metro stations are frequently located in densely populated and highly urbanized zones that are subject to elevated levels of air pollution, noise, and heat (69). These adverse environmental exposures may offset the physical activity benefits that are typically associated with public transit use (70, 71). Moreover, the density of metro stations may function as a proxy for urban intensity and traffic-related pollutant exposure rather than reflecting improved accessibility in itself, or it may indicate the spatial clustering of higher-risk populations in transit-rich areas. Besides, proximity to arterial roads was also found to be associated with increased stroke mortality, particularly in socioeconomically disadvantaged neighborhoods. In such contexts, heavy traffic, vehicular emissions, and excessive noise contribute to adverse cardiovascular outcomes through pathways involving systemic inflammation and oxidative stress (72). Taken together, these findings suggest that expanding transit infrastructure without simultaneously addressing broader environmental quality concerns may fail to achieve the intended cardiovascular health benefits.

In contrast, the presence of sidewalks exhibited an unexpected positive association with poorer cardiovascular outcomes, diverging from the prevailing literature that typically identifies sidewalks as facilitators of walkability and active living. One plausible explanation lies in the distinctive urban morphology of high-density Chinese megacities (73). While wide sidewalks may signal infrastructural readiness for pedestrian activity, they frequently adjoin multi-lane arterial roads characterized by heavy traffic volumes, suboptimal pedestrian-scale design, and insufficient visual or physical buffers from vehicular flows. These conditions may foster perceptions of unsafety, discomfort, and environmental stress, particularly among older adults with mobility limitations. Furthermore, sidewalks in certain districts may suffer from underutilization or inadequate maintenance, diminishing their functional contribution to physical activity promotion. Similar findings have been reported in Seoul (34), where conventional walkability indicators failed to translate into increased walking due to adverse environmental conditions. Consequently, the health implications of sidewalk provision should be interpreted within a broader contextual framework that incorporates aesthetic, environmental, and behavioral mediators, rather than as a unidimensional infrastructure metric.

This study has several limitations. First, as an ecological cross-sectional analysis, it cannot establish causal relationships between built environment characteristics and stroke mortality. Future research should adopt more rigorous designs, such as quasi-experimental or longitudinal approaches, to better evaluate the causal impact of built environment features on cardiovascular health. Second, due to data limitations, this study was unable to control for key individual-level factors such as education, occupation, income, medical history, physical activity, and family history, as well as potential environmental factors including meteorological variables (e.g., wind speed and temperature). Future studies should integrate individual-level health data with environmental exposure information to enhance the explanatory power and scientific validity of the findings. Third, differences in urban contexts across studies and geographic variations between countries lead to distinct lifestyles and living environments. In particular, within Western contexts, significant inequalities exist between the Global North and South, where cities in Latin America and the Caribbean often face greater vulnerability in terms of living conditions, urban infrastructure, and socioeconomic factors. Therefore, future research could consider comparative analyses among Chinese or Western cities or districts with similar size and urban structures to verify potential convergences in the evidence and further enrich the empirical understanding of the relationship between the built environment and cardiovascular health.

## 6 Conclusion

This study offers new empirical evidence on the associations between micro-scale built environment characteristics and cardiovascular health among older adults, with a particular focus on socioeconomic disparities. The findings show that certain urban design features, including high residential building density, excessive residential land coverage, limited greenery, proximity to major roads, and a greater number of metro stations, are associated with poorer cardiovascular health outcomes, especially among older adults living in socioeconomically disadvantaged neighborhoods. In contrast, greater availability of green space and enhanced visual openness, such as visible sky and streetscape greenery, are linked to better cardiovascular health, particularly in more affluent areas.

Based on the findings of this study, the following planning strategies are proposed to improve cardiovascular health outcomes among older adults, with particular attention to addressing socioeconomic disparities. Urban planning should prioritize the creation and equitable distribution of high-quality green spaces, especially in socioeconomically disadvantaged neighborhoods. Increasing access to parks, vegetation, and visually open environments can help mitigate environmental stressors such as air pollution and psychosocial strain. Incorporating natural elements like trees and visible sky into neighborhood design may encourage outdoor activity, reduce stress, and promote recovery, all of which contribute to improved cardiovascular health. Moreover, planners should adopt human-scaled, mixed-use development strategies that reduce land-use homogenization and improve access to essential services. This includes integrating housing, healthcare, retail, and recreational spaces within walkable neighborhoods to reduce reliance on motorized transport. Special attention should be given to streetscape design by incorporating greenery, shade, and traffic-calming features to ensure a safe and comfortable pedestrian environment, particularly for older adults. Additionally, buffer zones and vegetation barriers should be considered near major roads and transit corridors to reduce residents’ exposure to noise and air pollution. These evidence-based strategies can support active aging and help build more inclusive, healthy, and resilient urban communities. By aligning urban development strategies with public health objectives, particularly for aging and vulnerable populations, these evidence-based approaches can help create healthier, more resilient, and more equitable cities.

## Data Availability

The original contributions presented in the study are included in the article/supplementary material, further inquiries can be directed to the corresponding authors.

## Footnotes

1
http://lbsyun.baidu.com/

2
https://www.openstreetmap.org/
